# BRCA1-IRIS overexpression promotes and maintains the tumor initiating phenotype: implications for triple negative breast cancer early lesions

**DOI:** 10.18632/oncotarget.14357

**Published:** 2016-12-29

**Authors:** Abhilasha Sinha, Bibbin T. Paul, Lisa M. Sullivan, Hillary Sims, Ahmed El Bastawisy, Hend F. Yousef, Abdel-Rahman N. Zekri, Abeer A. Bahnassy, Wael M. ElShamy

**Affiliations:** ^1^ Cancer Institute, University of Mississippi Medical Center, Jackson, MS, USA; ^2^ Department of Molecular Biology and Biophysics, University of Connecticut Health Center, Farmington, CT, USA; ^3^ Department of Pathology, University of Mississippi Medical Center, Jackson, MS, USA; ^4^ Medical Oncology, National Cancer Institute, Cairo University, Cairo, Egypt; ^5^ Cytogenetics and Molecular Genetics, National Cancer Institute, Cairo University, Cairo, Egypt; ^6^ Virology and Immunology, National Cancer Institute, Cairo University, Cairo, Egypt; ^7^ Molecular Pathology and Cytogenetics, National Cancer Institute, Cairo University, Cairo, Egypt

**Keywords:** breast cancer, BRCA1-IRIS, metastasis, early lesion, TNBC

## Abstract

Tumor-initiating cells (TICs) are cancer cells endowed with self-renewal, multi-lineage differentiation, increased chemo-resistance, and in breast cancers the CD44^+^/CD24^-^/ALDH1^+^ phenotype. Triple negative breast cancers show lack of BRCA1 expression in addition to enhanced basal, epithelial-to-mesenchymal transition (EMT), and TIC phenotypes. BRCA1-IRIS (hereafter IRIS) is an oncogene produced by the alternative usage of the BRCA1 locus. IRIS is involved in induction of replication, transcription of selected oncogenes, and promoting breast cancer cells aggressiveness. Here, we demonstrate that IRIS overexpression (IRISOE) promotes TNBCs through suppressing BRCA1 expression, enhancing basal-biomarkers, EMT-inducers, and stemness-enforcers expression. IRISOE also activates the TIC phenotype in TNBC cells through elevating CD44 and ALDH1 expression/activity and preventing CD24 surface presentation by activating the internalization pathway EGFR→c-Src→cortactin. We show that the intrinsic sensitivity to an anti-CD24 cross-linking antibody-induced cell death in membranous CD24 expressing/luminal A cells could be acquired in cytoplasmic CD24 expressing IRISOE TNBC/TIC cells through IRIS silencing or inactivation. We show that fewer IRISOE TNBC/TICs cells form large tumors composed of TICs, resembling TNBCs early lesions in patients that contain metastatic precursors capable of disseminating and metastasizing at an early stage of the disease. IRIS-inhibitory peptide killed these IRISOE TNBC/TICs, *in vivo* and prevented their dissemination and metastasis. We propose IRIS inactivation could be pursued to prevent dissemination and metastasis from early TNBC tumor lesions in patients.

## INTRODUCTION

Breast cancer classification based on gene expression profile showed at least 5 distinct subtypes: luminal A, luminal B, normal breast-like, human epidermal growth factor receptor 2-positive, and triple-negative/basal-like [1]. Triple-negative breast cancer (TNBC) accounts for 15-20% of all breast cancers and lacks oestrogen receptor (ER), progesterone receptor (PR) expression, and human epidermal growth factor receptor 2 (HER2) amplification. Despite this simple definition, TNBCs are a heterogeneous group of aggressive tumors with highest relapse rates, poorest outcomes, shortest overall survival in the metastatic setting compared to other subtypes, and most importantly, a complete paucity of effective therapies [2].

TNBCs show a basal-like molecular profile [3], including overexpression of basal-biomarkers (e.g., CK5, CK14, CK17, and EGFR [1, 4, 5]). Closely associated with the induction of the basal phenotype in TNBCs is the acquisition of epithelial-to-mesenchymal transition (EMT), which is most likely involved in their initial steps of invasion and metastasis [6, 7], and drug resistance [8]. TNBCs clinical and pathologic features overlap with hereditary BRCA1 related breast cancers [9]. in fact, TNBCs are enriched for germline BRCA1 mutations, and women who carry a BRCA1 mutation typically develop TNBCs [10]. Both BRCA1-related cancers and TNBCs show induction of the tumor-initiating cells (TICs) phenotype. Indeed, basal-like carcinomas originate from mammary stem cells and physiological progenitors within the normal female breast share immunohistochemical features with basal-like breast cancers [1, 11].

TICs in breast cancer are CD44^+^CD24^-^ cells [12–16]. CD44 is a cell surface receptor expressed by many cell types, including breast cancer stem cells that interacts with a variety of effectors [17] and initiates many regulatory mechanisms related to adhesion, cell proliferation, migration, invasiveness, and chemo-resistance [18, 19]. CD24 is a small, heavily glycosylated cell surface receptor frequently overexpressed in a wide range of carcinomas, including breast carcinomas [20–22]. TICs have the ability to self-renew, grow as mammospheres, initiate tumor formation in mice [11], differentiate into a variety of cell types, and exhibit resistance to chemo-/radio-therapies [19]. TNBC patients have the highest rates of early recurrence, which is thought to be due to the TICs in them. Indeed, preclinical and clinical evidences do show chemo- and radiotherapy select for TICs in these tumors. Although several recent studies suggested CD24 expression could serve as a marker for adverse prognosis in certain tumors [23–25], low surface expression seems to enhance aggressiveness in breast cancer TICs [13]. Indeed, scanning electron microscopy showed CD44^+^CD24^-/low^ breast cancer cells at the tumor invasive protrusions [26].

These protrusions usually called invadopodia (a hallmark of EMT in breast tumors) are specialized actin polymerization-driven structures used to degrade and possibly invade through the extracellular matrix (ECM) during invasion and metastasis [27]. Epidermal growth factor receptor (EGFR) is frequently overexpressed in TNBCs and progenitor cells in BRCA1 mutation carriers [28]. In these cells high level of activated c-Src are usually associated with membranous EGFR [29]. The EGFR→Src→cortactin signaling pathway is thought to be critical for invadopodia formation in TNBCs [30, 31]. However, this pathway is proposed to be involved in membrane proteins endocytosis as well, especially in EGFR overexpressing cells [32–34].

BRCA1-IRIS (hereafter IRIS) is a recently discovered oncogene produced by the alternative usage of the BRCA1 locus that contains the first 11 exons of BRCA1 *plus* a 34 amino acid read-through from intron 11 [35]. IRIS overexpression (hereafter IRISOE) promotes endoreplication [35] and the transcription of selected oncogenes, e.g., cyclin D1 and EGFR [36, 37]. In breast cancers, IRISOE correlates with poor prognosis, aggressive features, and the basal phenotype [38]. *In vitro*, IRISOE promotes migration and invasiveness of breast and ovarian cancer cells [36, 39]. *In vivo*, IRISOE promotes TNBCs formation [38, 40]. We designed a novel IRIS-inhibitory peptide that killed TNBC cells, *in vitro* and induced TNBC tumor regression, *in vivo* [36].

The old view that metastatic breast cancer cells are rare, late arising cells due to progressive accumulation of mutations has been challenged recently [41]. The new view proposes that metastatic precursors with a TIC phenotype do exit within early tumor lesions [42–44]. We investigated whether IRISOE TNBC cells show TIC phenotype and whether they are able to disseminate and metastasize from early lesions. We show IRISOE suppresses BRCA1 expression, enhances basal-biomarkers, EMT-inducers, and stemness-enforcers expression, and promotes the TIC phenotype. Additionally, using pre-clinical animal models and human clinical specimens, we confirmed IRISOE TNBC/TICs are able to disseminate from early tumor lesions and metastasize. Finally, we show that IRIS-inhibitory peptide kills TNBC tumors, *in vivo* by specifically depleting their TICs.

## RESULTS

To experimentally define whether IRISOE drives the TNBC phenotype in breast cancer cells, we analyzed IRISOE association with the known criteria for TNBCs; namely lack of BRCA1 expression, enhanced basal-biomarkers, EMT-inducers, stemness-enforces expression, and TIC phenotype.

### IRISOE suppresses BRCA1 expression in breast cancer cells

**Figure 1:**
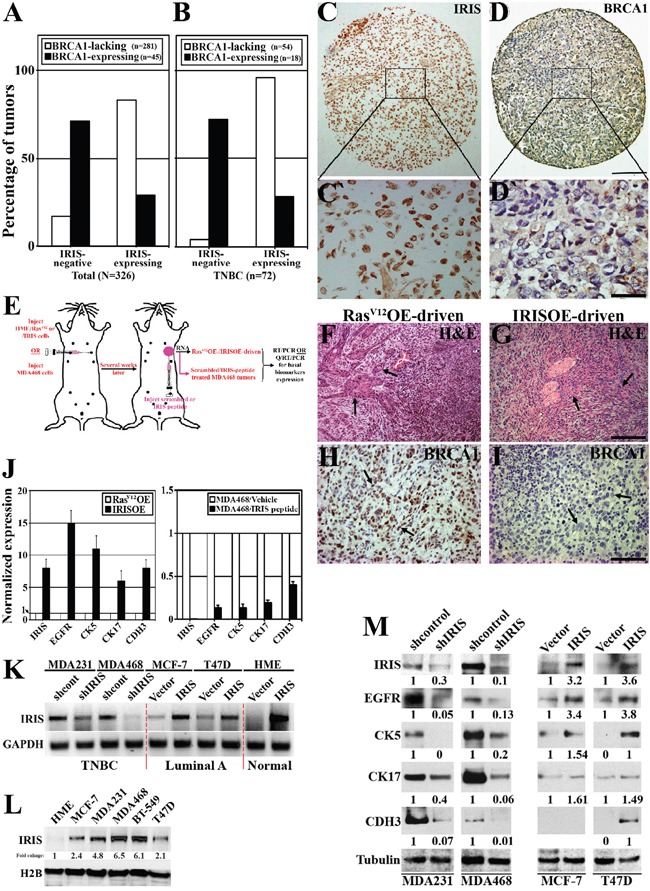
IRISOE suppresses BRCA1 expression and enhances basal-biomarkers expression in breast cancer cells Immunohistochemical analysis of IRIS and BRCA1 expression in a cohort of breast tumor (all subtypes, n=326, **A**), or a sub-cohort of TNBC tumors (n=72, **B**). Representative images of IRISOE (**C**, and larger magnification C`) associated with lack of BRCA1 expression (**D**, and larger magnification D`) in a TNBC tumor sample. Scale bars: 300μm in C and D, and 50μm in C` and D`. E. Schematic of the strategy used to generate RasV12OE-/IRISOE-driven or MDA468 + scrambled/MDA468 + IRIS inhibitory peptide orthotopic mammary tumors in SCID/Nu/Nu mice, followed by tumor and RNA isolation and basal-biomarkers expression analysis. H&E (F and G) and BRCA1 (H and I) staining on RasV12-driven or IRISOE-driven orthotopic mammary tumors, respectively generated as in (E). Scale bars: 200μm in F and G, and 100μm in H and I. J. real-time QRT/PCR analysis for the expression of IRIS and several basal-biomarkers mRNA in RasV12OE-driven or IRISOE-driven orthotopic mammary tumors (left), and MDA468 orthotopic mammary tumors after treatment with scrambled- or IRIS-inhibitory peptide (right). K. RT/PCR analysis of IRIS mRNA in naïve HME or the luminal cell lines; MCF7 and T47D before and after IRISOE and the TNBC cell lines; MDA231 and MDA468 before and after IRIS knockdown. L. Comparison of IRIS protein expression in the luminal A cell lines; MCF7, T47D, and the TNBC cell lines; MDA231, MDA468 and BT-549 compared to naïve HME cells. M. Western blot analysis for the expression of several basal-biomarkers in the TNBC cell lines; MDA231 and MDA468 before and after IRIS-silencing, and the luminal cell lines; MCF7 and T47D before and after IRISOE.

Our previous analysis of a large cohort of breast tumor samples (n>500) showed that IRISOE correlates with lack of BRCA1 expression [38]. To confirm this data, we immunohistochemically (IHC) stained adjacent sections from a breast cancer cohort (n=326, of all subtypes) with a mouse monoclonal anti-IRIS antibody raised against the intron 11 domain of IRIS (does not cross react with BRCA1 [35]) and a mouse monoclonal anti-BRCA1 antibody raised against the very C-terminal sequence of exon 24 of BRCA1 (does not cross react with IRIS [35]) on adjacent sections. About 86% (281/326) of the tumors in this cohort were BRCA1-lacking (i.e. show no protein expression); whereas, 14% (45/326) were BRCA1-positive (expressed normal level BRCA1 protein). Within the BRCA1-lacking group, 17% (47/281) were IRIS-negative (express level in normal cells), while 83% (234/281) were IRIS-expressing (i.e. IRISOE = express ≥ 2fold above level in normal cells, white bars, Figure 1A). Conversely, within the BRCA1-expressing group, 71% (32/45) were IRIS-negative, while 29% (13/45) were IRISOE tumors (black bars, Figure 1A).

Within this cohort, a sub-cohort of TNBC tumors (n=72) contained 75% (54/72) BRCA1-lacking and 25% (18/72) BRCA1-positive tumors. Within the TNBC/BRCA1-lacking group, only 4% (2/54) were IRIS-negative, while 96% (52/54) were IRISOE tumors (white bars, Figure 1B). See example of an IRISOE TNBC tumor (Figure 1C and larger image in Figure 1C`) that show no BRCA1 expression (Figure 1D and larger image in Figure 1D`). Conversely, within the TNBC/BRCA1-expressing group, 72% (13/18) were IRIS-negative tumors and 28% (5/18) were IRIS-positive (black bars, Figure 1B).

We recently used naïve HME (BRCA1-expressing) cells infected with Ras^V12^ (positive control) or inducible IRIS alleles to generate orthotopic mammary tumors in SCID mice (Figure 1E, [38]). Ras^V12^OE-driven tumors were circumscribed, non-invasive, low grade with excessive glandular structures (see arrows, Figure 1F), expressed epithelial biomarkers, and ERα suggesting their luminal phenotype [38]. In contrast, IRISOE-driven tumors were invasive, high grade with prominent spindle cell component (see arrows, Figure 1G), expressed high-level EMT inducers, but no ERα suggesting their TNBC phenotype [38]. Interestingly, like human tumors, compared to Ras^V12^OE-driven orthotopic tumors (see arrows, Figure 1H), IRISOE-driven tumors were completely devoid of BRCA1 expression (see arrows, Figure 1I, [38]). Taken together, these data show that in breast cancers in human or in animal models IRISOE tumors show lack of BRCA1 expression. Mechanism(s) responsible for this suppression is still unknown.

### IRISOE promotes basal-biomarkers expression in breast cancer cells

Total RNAs were isolated from Ras^V12^OE-driven or IRISOE-driven orthotopic mammary tumors (Figure 1E), and orthotopic mammary tumors generated using the TNBC cell line; MDA-MB-468 (hereafter MDA468) that were intratumorally treated with a scrambled or IRIS-inhibitory peptide (Figure 1E). Detail analysis of the structure and specificity of this inhibitory peptide *in vitro* and *in vivo* was reported recently [36, 39]. Noteworthy here, this inhibitory peptide had no effect on BRCA1 expression/activity.

Real-time QRT/PCR was used to compare expression of the basal biomarkers: *EGFR, cytokeratin 5 (CK5), CK17 and p-cadherin (CDH3) mRNA* [9] in Ras^V12^OE- vs. IRISOE-driven tumors, and in scrambled peptide vs. IRIS-inhibitory peptide treated orthotopic MDA468 tumors (Figure 1E). Compared to normalized expressions in Ras^V12^OE-driven tumors (white bars, Figure 1J, left), the expressions of these basal biomarkers *mRNA* in IRISOE-driven orthotopic mammary tumors were significantly higher (black bars, Figure 1J, left). Moreover, compared to normalized expressions in scrambled peptide treated MDA468 tumors (white bars, Figure 1J, right), the expressions of these basal biomarkers *mRNA* in IRIS-inhibitory peptide treated MDA468 tumors were significantly lower (black bars, Figure 1J, right).

Furthermore, compared to naïve human mammary epithelial (HME) cells, expression of IRIS *mRNA* (Figure 1K) and protein (Figure 1L) increase slightly in luminal A cell lines (e.g., MCF7 and T47D), while significantly in TNBC cell lines (e.g., MDA-MB-231 [hereafter MDA231], MDA468, and BT-549). IRIS specific shRNA (shIRIS) significantly decreased *IRIS mRNA* (Figure 1K) and protein (Figure 1M) expression in MDA231 and MDA468 to luminal A cell line levels (Figure 1K and 1C), which led to significant decrease in EGFR, CK5, K17 and CDH3 expression in both cell lines (Figure 1M, left). IRIS overexpression (hereafter IRISOE) in MCF7 and T47D to TNBC cell lines level (Figure 1K and 1M) significantly increased EGFR, CK5, CK17, and CDH3 expression in both cell lines (Figure 1M, right). Taken together, these data show that IRISOE enhances basal-biomarkers expression in breast cancer cells, *in vitro* and *in vivo*, although mechanism(s) responsible for this enhancement is still unknown.

### IRISOE promotes EMT-inducers expression in breast cancer cells

**Figure 2:**
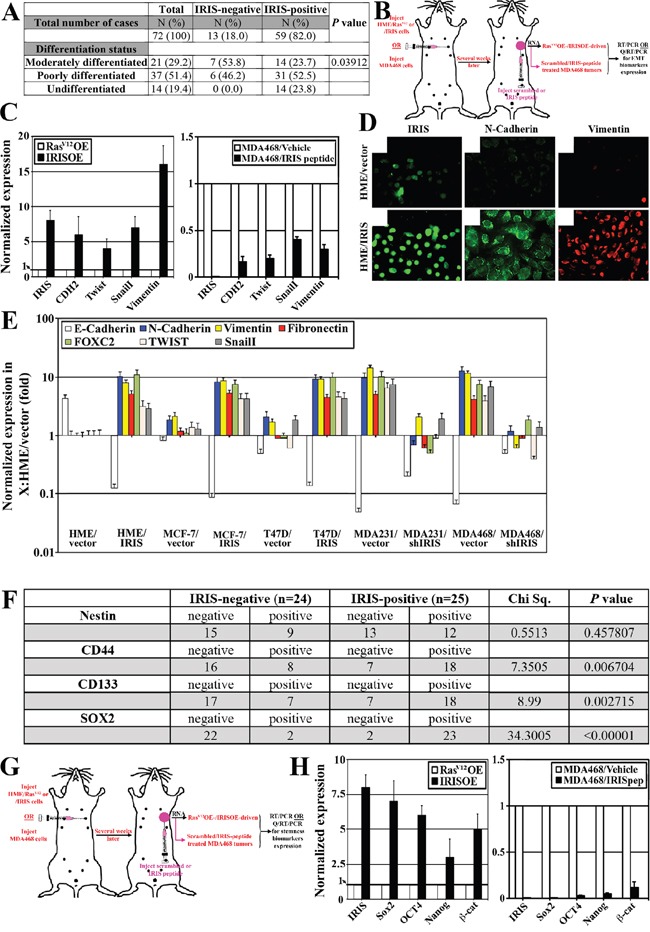
IRISOE promotes expression of EMT-inducers and stemness-enforcers in breast cancers A. Association analysis between IRIS expression and the differentiation status of TNBC tumors (n=72). B. Schematic of the strategy used to generate RasV12OE-/IRISOE-driven or MDA468 + scrambled/MDA468 + IRIS inhibitory peptide orthotopic mammary tumors in SCID/Nu/Nu mice, followed by tumor and RNA isolation and EMT-inducers expression analysis. C. Real-time QRT/PCR analysis showing the expression of IRIS and several EMT-inducers mRNAs in RasV12OE-driven or IRISOE-driven orthotopic mammary tumors (left), and MDA468 orthotopic mammary tumors after treatment with scrambled- or IRIS-inhibitory peptide (right), generated as in (B). D. Immunofluorescence analysis of IRIS, N-cadherin and vimentin expression in naïve (uppers) or IRISOE (lowers) HME cells. E. Real time QRT/PCR of indicated mRNAs in naïve/IRISOE HME cells, vector/IRIS transfected MCF7 or T47D, and shcontrol/shIRIS transfected MDA231 or MDA468. F. Association analysis between IRISOE and the expression of several TIC-biomarkers in a cohort of locally advanced breast cancer patients (n=49, from Egyptian NCI) conducted using real-time QRT/PCR. G. Schematic of the strategy used to generate RasV12OE-/IRISOE-driven or MDA468 + scrambled/MDA468 + IRIS inhibitory peptide orthotopic mammary tumors in SCID/Nu/Nu mice, followed by tumor and RNA isolation and stemness-enforcers expression analysis. H. Real-time QRT/PCR analysis for the expression of IRIS and the stemness-enforcers mRNAs expression in RasV12OE-driven or IRISOE-driven orthotopic mammary tumors (left), and MDA468 orthotopic mammary tumors after treatment with scrambled- or IRIS-inhibitory peptide (right), generated as (G).

The TNBC cohort described above (n=72) was also analyzed for tumor morphology. Within this cohort, 18% (13/72) of the tumors are IRIS-negative, while 82% (59/72) of the tumors are IRIS-positive (see above for definition). Within this cohort, 29% (21/72) of the tumors were moderately differentiated, 33% (7/21) of those were IRIS-negative, and 67% (14/21) were IRIS-positive tumors (Figure 2A), 51% (37/72) of the tumors were poorly differentiated, 16% (6/37) of those were IRIS-negative, and 84% (31/37) were IRIS-positive tumors (Figure 2A), while 19% (14/72) of the tumors were undifferentiated, and all were IRIS-positive tumors (*p=0.03912*, Figure 2A).

Real-time QRT/PCR was also used to compare expression of the EMT inducers: *N-cadherin (CDH2), vimentin (Vim), Snail and Twist mRNA* in Ras^V12^OE- vs. IRISOE-driven tumors, and in scrambled peptide vs. IRIS-inhibitory peptide treated orthotopic MDA468 tumors (Figure 2B). Compared to Ras^V12^OE-driven tumors (white bars, Figure 2C, left), the expressions of these EMT-inducers *mRNA* were significantly higher in IRISOE-driven tumors (black bars, Figure 2C, left). By contrast, the high expressions level of these EMT-inducers *mRNAs* detected in scrambled peptide treated orthotopic MDA468 tumor samples (white bars, Figure 2C, right) were significantly decreased in IRIS-inhibitory peptide treated MDA468 tumors (black bars, Figure 2C, right).

IRISOE in naïve HME or luminal A; MCF7 and T47D cells initiates/enhances expression of the EMT biomarkers; CDH2, Vim, fibronectin (FIN), FOXC2, Twist and Snail (Figure 2D and 2E), whereas IRIS silencing significantly decreased their expression in the high expressing TNBC cell lines (MDA231 and MDA468, Figure 2E). In contrast, expression of the epithelial biomarker E-cadherin (CDH1) was significantly decreased in naïve HME, MCF7, or T47D cell lines upon IRISOE, and its expression was restored when IRIS was silenced in MDA231 and MDA468 (Figure 2E). In fact, morphological changes associated with EMT are observed in T47D cells upon IRISOE (compare Supplementary Figure 1B to 1A), whereas morphological changes associated with mesenchymal to epithelial transition (MET) are observed in MDA468 cells upon IRIS silencing (compare Supplementary Figure 1D to 1C). Taken together, these data suggest IRISOE through enhancing EMT-inducers expression promotes de-differentiation/EMT in TNBC cells/tumors, *in vitro* and *in vivo*, although mechanism(s) responsible for that is unknown.

### IRISOE promotes expression of stemness-enforcers in breast cancer cells

Recently, we showed that IRISOE elevates expression of the stemness enforcers: Sox2, Oct4, and Nanog in naïve HME and its silencing reduces them in TNBC cell lines [36]. To expand this association into human breast tumor samples, a cohort of locally advanced breast tumors (n=49) collected at the Egyptian NCI (see Material and Methods for details) was analyzed for *IRIS mRNA* expression using real-time QRT/PCR. This analysis showed 49% (24/49) of the tumors were IRIS-negative, whereas 51% (25/49) of the tumors were IRIS-positive (see above for definition, Figure 2F). Expressions of the stemness biomarkers; nestin, CD44, CD133, and Sox2 were also investigated in these tumors using IHC. Among the IRIS-negative tumors, 63% (15/24) were negative and 37% (9/24) were positive for nestin, whereas among the IRIS-positive tumors, 52% (13/25) were negative and 48% (12/25) were positive for nestin (Chi Sq. 0.5513, *p=0.457807*, Figure 2F). Among the IRIS-negative tumors, 67% (16/24) were negative and 33% (8/24) were positive for CD44, while among the IRIS-positive tumors, 28% (7/25) were negative and 72% (18/25) were positive for CD44 (Chi Sq.=7.3505, *p=0.006704*, Figure 2F). Among the IRIS-negative tumors, 71% (17/24) were negative and 29% (7/24) were positive for CD133, while among the IRIS-positive tumors, 28% (7/25) were negative and 72% (18/25) were positive for CD133 (Chi Sq.=8.99, *p=0.002715*, Figure 2F). Finally, among the IRIS-negative tumors, 92% (22/24) were negative and only 8% (2/24) were positive for Sox2, while among the IRIS-positive tumors, only 8% (2/25) were negative and 92% (23/25) were positive for Sox2 (Chi Sq.=34.3005, *p<0.00001*, Figure 2F).

Real-time QRT/PCR was also used to compare expression of the stemness-enforcers: *Sox2, Oct4, Nanog and β-catenin mRNAs* in Ras^V12^OE- vs. IRISOE-driven tumors, and scrambled peptide vs. IRIS-inhibitory peptide treated orthotopic MDA468 tumors (Figure 2G). Compared to Ras^V12^OE-driven tumors (white bars, Figure 2H, left), the expressions of these stemness-enforcers *mRNAs* in IRISOE-driven tumors were significantly increased (black bars, Figure 2H, left). By contrast, the high level expressions of these stemness-enforcers *mRNAs* detected in scrambled peptide treated orthotopic MDA468 tumors (white bars, Figure 2H, right) were significantly decreased when tumors were treated with IRIS-inhibitory peptide (black bars, Figure 2H, right). Taken together, these data suggest high correlation between IRISOE and high expression of the breast cancer stem cell biomarkers (e.g., CD44 and CD133) and the stemness-enforcers (e.g., Sox2, Oct4, Nanog and β-catenin) in locally advanced breast cancers, pre-clinical animal models, and *in vitro* although mechanism(s) responsible for that are still unknown.

### IRISOE establishes the TIC/CD44^+^CD24^-/low^ ALDH1^+^ phenotype in breast cancer cells

**Figure 3:**
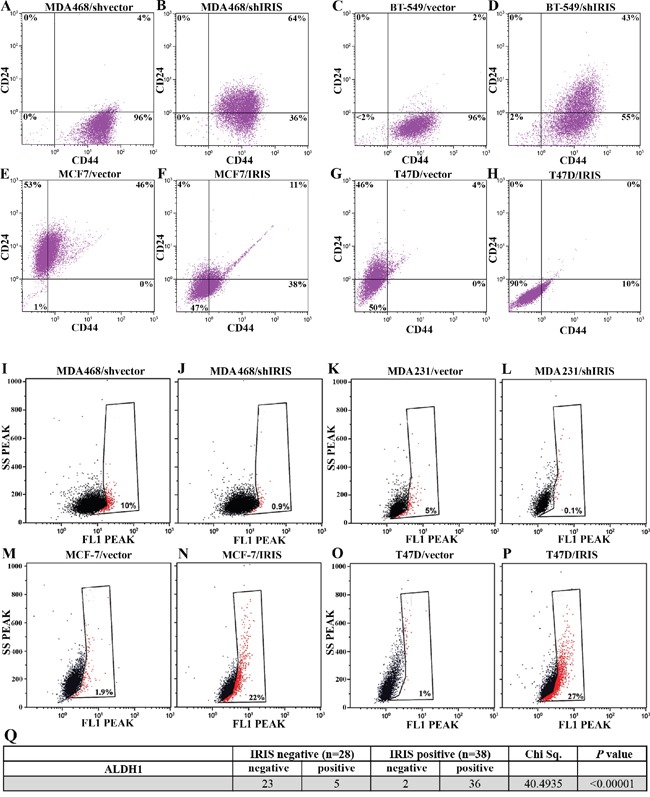
IRISOE drives the TIC phenotype FACS analysis for surface CD44 and CD24 double-staining (for 1h) populations within shcontrol- A., or shIRIS- B. expressing MDA468 cells, shcontrol- C., or shIRIS- D. expressing BT-549 cells, vector E., or IRIS- F. overexpressing MCF7, vector- G., or IRIS- H. overexpressing T47D cells. FACS analysis showing ALDH1+ (red) compared to ALDHA- (black) populations in shcontrol- I., or shIRIS- J. expressing MDA468 cells, shcontrol- K., or shIRIS- L. expressing MDA231 cells, vector M., or IRIS- N. overexpressing MCF7, vector- O., or IRIS- P. overexpressing T47D cells. Q. Association analysis of IRIS and the expression of ALDH1 in a cohort of metastatic breast cancer patients (n=66, from Egyptian NCI) conducted using real-time QRT/PCR.

To further establish the association between IRISOE and the TIC phenotype in breast cancer cells, FACS analysis of non-permeabilized cells (to detect surface proteins only) using FITC-CD44 and PE-CD24 antibodies was performed. In 2 TNBC cell lines (parental MDA468 and parental BT-549), 96% of the cells showed the TIC/CD44^+^CD24^-^ profile (Figure 3A and 3C, respectively). IRIS silencing converted 60% of MDA468 (compare Figure 3B-3A) and 41% of BT-549 (compare Figure 3D-3C) cells from TICs/CD44^+^CD24^-^ cells into non-TICs/CD44^+^CD24^+^ cells. Noteworthy, the distribution of the CD44 signal in the silenced cells shifted to the left (compare Figure 3B-3A and Figure 3D-3C) suggesting a decrease in the number of CD44 receptors/cell, as well upon IRIS silencing in TNBC cells. Conversely, parental MCF7 and T47D cells showed no cells with the TIC/CD44^+^CD24^-^ profile (Figure 3E and 3G). IRISOE converted 38% of MCF7 cells (compare Figure 3F-3E) and 10% of T47D cells into TIC/CD44^+^CD24^-^ (compare Figure 3H-3G), suggesting enhanced CD44 expression and decrease CD24 expression/surface presentation.

Moreover, ALDH1 activity was significantly decreased when IRIS is silenced in MDA468 cells (compare Figure 3J-3I) and MDA231 cells (compare Figure 3L-3K), while significantly increased by IRISOE in MCF-7 cells (compare Figure 3N and 3M) and T47D cells (compare Figure 3P and 3O). In an effort to explain this effect, immunofluorescence (IF) analysis was used to measure the expression level of ALDH1/ALDH1A1 in the different conditions. The high ALDH1 expression detected in TNBC MDA468 cells (Supplementary Figure 2A), was significantly decreased upon IRIS silencing (Supplementary Figure 2B). In contrast, the low expression level detected in luminal A T47D cells (Supplementary Figure 2C) was significantly increased upon IRISOE (Supplementary Figure 2D).

To further support these observations using human samples, a second cohort of metastatic breast cancer tumors (n=66) also collected at the Egyptian NCI was analyzed for the expression of ALDH1 protein using IHC. Quantitative real-time RT/PCR showed that 42% (28/66) of this cohort were IRIS-negative tumors, while 58% (38/66) were IRIS-positive tumors (see above for definition). Among the IRIS-negative tumors, 82% (23/28) were negative, and 18% (5/28) were positive for ALDH1 expression. Among the IRIS-positive group, 5% (2/38) were negative, and 95% (36/38) were positive for ALDH1 expression (Chi Sq.=40.4935, *p<0.00001* Figure 3Q). Taken together, these data suggest that IRISOE establishes the TIC phenotype in TNBC cells by enhancing CD44 expression, enhancing ALDH1 expression/activity, and suppressing CD24 expression/access to the cell surface.

### IRISOE maintains the TIC/CD44^+^CD24^-/low^ phenotype in breast cancer cells: Candidate signaling pathway involved in CD24 membrane exclusion in TNBC cells

An intriguing possibility from the above data is that IRISOE is able to suppress CD24 transcription, stability, or membrane localization in TNBC cells. Although our unpublished data indeed show a negative regulation by IRISOE on CD24 transcription through increasing Twist expression [45], this could not explain the fact that many primary IRISOE/TNBC tumors and tumor cell lines maintain cytoplasmic CD24 expression (see discussion). Therefore, we investigated the effect of IRISOE on CD24 membrane localization in TNBC/TICs.

Membranous and cytoplasmic proteins isolated from MDA468 cells expressing shcontrol or shIRIS + siluciferase (siLuc) or shIRIS + siCD24, and from T47D cells expressing vector or IRIS cDNA were probed for CD24 expression. CD24 is a GPI-anchored membrane glycoprotein possesses several potential *N*- and *O*-glycosylation sites, which are extensively used so that strikingly high apparent molecular weights are observed ranging from 28-70kDa [46]. In the cytoplasm the protein appears as a single ∼30kDa protein.

**Figure 4:**
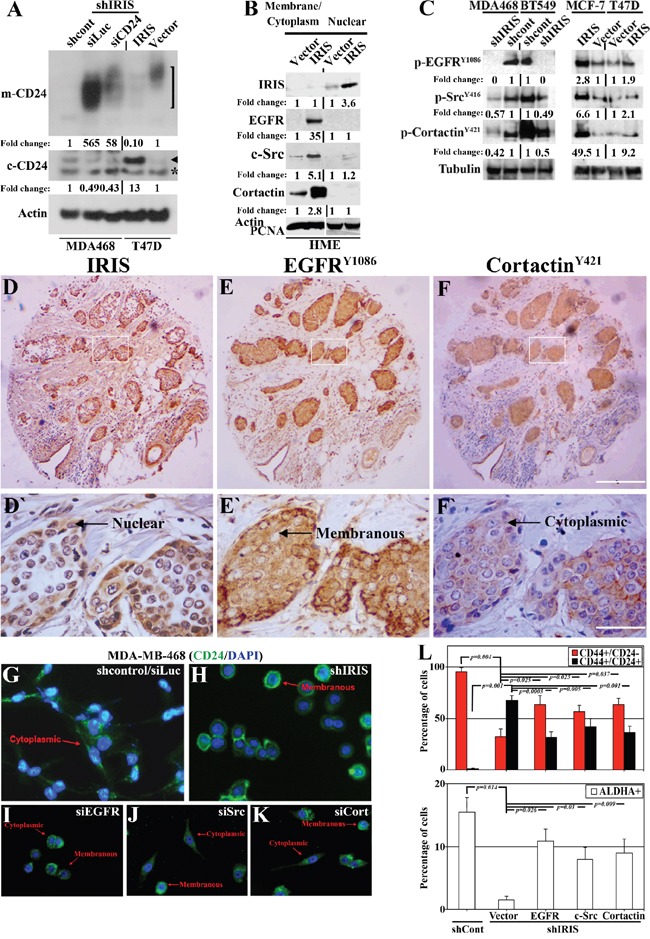
Potential signaling pathway promotes CD24 cytoplasmic confinement in IRISOE TNBC cells A. Western blot analysis for m-CD24 (open bracket, upper) or c-CD24 (arrowhead, lower, note asterisk indicates a non-specific band) in shcontrol- or shIRIS-expressing MDA468 cells expressing or not siCD24 (left), as well as vector or IRISOE T47D cells (right). B. Western blot analysis for the expression of nuclear IRIS and cytoplasmic/membranous EGFR, c-Src, and cortactin in naïve or IRISOE HME cells. C. Western blot analysis for the expression of p-EGFRY1086, p-SrcY416 and p-cortactinY421 in sonicates from shcontrol- or shIRIS-expressing MDA468 and BT-549 cells (left) and parental or IRIS-expressing MCF7 and T47D cells (right). The expression of IRIS D and D`. EGFRY1086 E and E`. and cortactinY421 F and F`. detected using IHC in a TNBC tumor sample. G-K. Immunofluorescence analysis for the expression of CD24 in shcontrol- (G), shIRIS-expressing (H), transiently siEGFR (I), siSrc (J), or sicortactin (K) transfected MDA468 cells. L. FACS analysis showing the percentage of CD44+/CD24- and CD44+/CD24+ (upper) and ALDHA+ (lower) in shcontrol-, or shIRIS-expressing MDA468 cells without and with EGFR, Src, or cortactin transient overexpression.

As expected, MDA468/shcontrol cells showed an exclusive cytoplasmic form of CD24 (c-CD24, see arrowhead in lane 1, lower panel, Figure 4A), the majority of which was re-directed to the membrane (m-CD24) upon IRIS silencing (see opened bracket in lane 2, upper panel, Figure 4A). CD24 silencing in IRIS-silenced cells abolished the expression of the m/c-CD24 (opened bracket and arrowhead, lane 3, Figure 4A). Conversely, vector-expressing T47D showed exclusively m-CD24 expression (opened bracket in lane 5, upper panel, Figure 4A) re-directed to the cytoplasm upon IRISOE (arrowhead in lane 4, lower panel, in Figure 4A). This was further confirmed *in vivo* through immunofluorescence (IF) analysis of MDA468 orthotopic tumors treated with scramble or IRIS-inhibitory peptide [36]. As expected exclusive c-CD24 expression in scrambled peptide treated (Supplementary Figure 3A) becomes mostly m-CD24 in IRIS-inhibitory peptide treated (Supplementary Figure 3B) MDA468 orthotopic mammary tumors (the non-uniform effect most likely is due to the intratumoral not systemic injection of the peptide).

The signaling pathway EGFR→Src→cortactin is implicated not only in invadopodia formation [30, 33], but also in surface proteins internalization [34, 47]. Gene expression microarray analysis showed that IRISOE significantly increased *EGFR*, *c-Src* and *EMS1 mRNAs* expression in naïve HME cells (unpublihed). Chromatin immunoprecipitation (ChIP) assay showed that IRIS protein is bound to *EGFR and EMS1* (*c-Src* promote is now under investigation) promoters in breast cancer cells and enhances their transcription (unpublished). Cytoplasmic/membranous (combined) and nuclear fractions from naïve and IRISOE HME cells were isolated. As expected IRISOE in naïve HME cells (nuclear fraction, Figure 4B) significantly increased expression of EGFR, c-Src, and cortactin (cytoplasmic/membranous fraction, Figure 4B).

Finally, analysis of cells sonicates showed that IRIS silencing significantly decreased the high levels of activated p-EGFR^Y1086^, p-Src^Y416^ and p-cortactin^Y421^ detected in the parental MDA468 (compare lane 1 to 2, Figure 4C, left), and BT-549 (compare lane 4 to 3, Figure 4C, left) TNBC cells. Most likely due to IRIS silencing decrease expression of the ligand EGF [36]. Conversely, IRISOE significantly increased the low levels of p-EGFR^Y1086^, p-Src^Y416^ and p-cortactin^Y421^ in the luminal A cell line: MCF7 (compare lane 2 to 1, Figure 4C, right) and T47D (compare lanes 4 to 3, Figure 4C, right), most likely through enhancing EGF expression in these cells [36].

To expand this data, the TNBC cohort (n=72) studied above was IHC stained with antibodies against IRIS, p-EGFR^Y1086^, or p-cortactin^Y421^ on adjacent sections. This analysis showed co-localization of nuclear IRIS (see representative image, Figure 4D and 4D`), membranous EGFR^Y1086^ (see representative image, Figure 4E and 4E`), and cytoplasmic cortactin^Y421^ (see representative image, Figure 4F and 4F`). Quantification of these stainings showed no cells where only one marker observed. Instead, many tumor cells stained with a pair of markers, and the majority stained with all three (Supplementary Figure 3C). Indeed, compared to 73.3% ± 12.7 of the cells showed IRIS + p-EGFR^Y1086^ + p-cortactin^Y421^ staining, 12.3% ± 9.4 of the cells showed IRIS + p-EGFR^Y1086^ staining (*p<0.00001*, Supplementary Figure 3C), or 14.4% ± 8.9 of the cells showed IRIS + p-cortactin^Y421^ staining (p<0.00001, Supplementary Figure 3C).

Consistent with the data presented in Figure 4A, IF analysis showed exclusive c-CD24 in MDA468/shcontrol cells (arrow in Figure 4G), re-surfaced as m-CD24 when IRIS is silenced (arrow in Figure 4H). Transfection of EGFR, c-Src, or cortactin siRNAs in the MDA468/shcontrol cells also re-directed the c-CD24 into m-CD24 expression in some but not all of the cells (likely depending on the siRNA transfection efficiency, see arrows in Figure 4I-4K). Additionally, the exclusive TIC/CD44^+^CD24^-^ profile documented for MDA468/shcontrol cells (see Figure 3A and Figure 4L, upper), and the high ALDH1^+^ profile (see Figure 3I and Figure 4L, lower) were significantly decreased when IRIS was silenced (see Figure 3B and Figure 4L, upper for the TIC/CD44^+^CD24^-^ profile, and Figure 3J and Figure 4L, lower for the ALDH1^+^ profile). Exogenous transient expression of EGFR, c-Src, or cortactin in MDA468/shIRIS cells partially restored the high TIC/CD44^+^CD24^-^ (Figure 4L, upper), and ALDH1^+^ (Figure 4L, lower) profiles. Taken together, these data suggest that IRISOE through enhancing the EGFR→c-Src→cortactin signaling pathway expression and activity maintains the invasive (enhancing invadopodia) and TIC (through CD24 membrane exclusion) phenotypes in TNBC cells, *in vitro* and *in vivo*.

### The intrinsic sensitivity in luminal cells to anti-CD24 cross-linking antibody can be acquired in TNBC cells through IRIS silencing

Anti-CD24 cross-linking antibody has been previously used to kill CD24-expressing leukemia cells, *in vitro* and *in vivo* [48, 49], which raises the interestingly clinical implication that inactivating IRIS could sensitize TNBC cells, *in vivo* to an anti-CD24 cross-linking antibody therapy.

**Figure 5:**
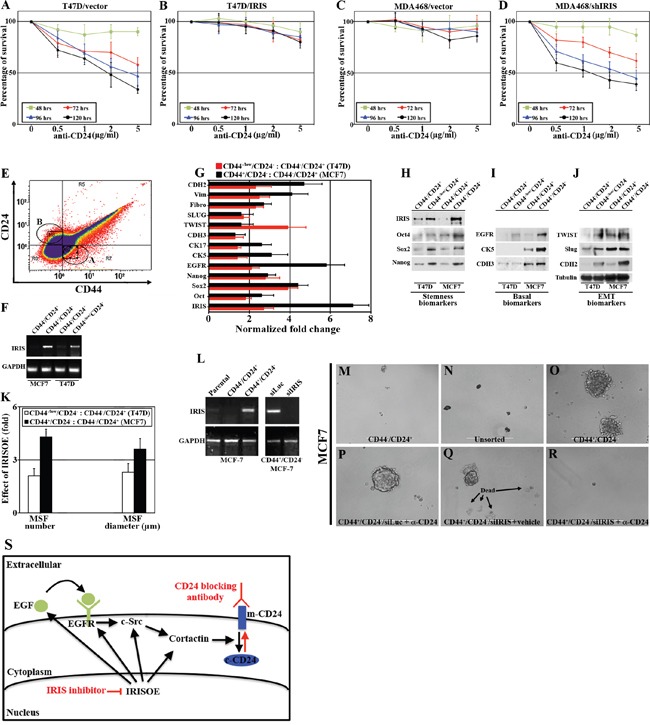
Intrinsic sensitivity in luminal A cancer cells to an anti-CD24 blocking antibody could be acquired in TNBC/TIC cells following IRIS inhibition Percentage of survival in vector- A. or IRIS- B. overexpressing T47D cells, as well as shcontrol- C. or shIRIS- D. expressing MDA468 cells following incubation with increasing concentration of anti-CD24 monoclonal antibody for the indicated times. E. FACS analysis of non-permeabilized MCF7 cells incubated with FITC-CD44 and PE-CD24 antibodies for only 20mins. F. Expression of IRIS mRNA in non-CD44-/CD24+ and TIC-CD44+/CD24- MCF7 cells, or non-CD44-/CD24+ and TIC-like-CD44-/CD24- T47D cells sorted as in (E). G. Real time QRT/PCR analysis for the expression of IRIS and the indicated basal-biomarkers, EMT-inducers, and stemness-enforcers mRNA in MCF7 TIC-CD44+/CD24- cells compared to non-CD44-/CD24+ cells, and T47D TIC-like-CD44-/CD24- cells compared to non-CD44-/CD24+ cells sorted as in (E). H-J. Western blot analysis for the expression of IRIS and the indicated basal-biomarkers, EMT-inducers, and stemness-enforcers non-CD44-/CD24+ and TIC-CD44+/CD24- MCF7 cells, or non-CD44-/CD24+ and TIC-like-CD44-/CD24- T47D cells sorted as in (E). K. Numbers and diameters of mammospheres developed by non-CD44-/CD24+ and TIC-CD44+/CD24- MCF7 cells, or non-CD44-/CD24+ and TIC-like-CD44-/CD24- T47D cells sorted as in (E). L. RT/PCR analysis of IRIS mRNA in parental, non-TIC and TIC MCF7 cells sorted as in (E) before and after IRIS silencing. Mammosphere formation by non-TIC M. parental unsorted N., TIC O. MCF7 cells before or after anti-CD24 blocking antibody + siLuc P. vehicle + siIRIS Q. or anti-CD24 blocking antibody + siIRIS R. treatment. S. Schematic presentation of our hypothesis of sensitizing TNBC/TIC cells to anti-CD24 blocking antibody through IRIS silencing/inhibition.

To test the efficacy of re-surfacing CD24 through IRIS silencing in eliminating TNBC/TIC cells using an anti-CD24 cross-linking antibody, equal number of vector- (i.e. IRIS^low^/m-CD24^high^) or IRIS- (i.e. IRIS^high^/m-CD24^low^) expressing T47D cells, and shcontrol- (i.e. IRIS^high^/m-CD24^low^) or shIRIS- (i.e. IRIS^low^/m-CD24^high^) expressing MDA468 cells were plated in the presence of increasing concentration (0, 0.5, 1, 2 and 5μg/ml added once) of the cross-linking anti-CD24 monoclonal antibody. At 48, 72, 96 or 120h later cells present in each well were manually counted and plotted. At 48h, no measurable loss of cell survival was detected at any concentration tested in all cultures (Figure 5A-5D). However, at 72h we found that compared to vehicle treated cells, a significant and gradual decrease of cell survival with increased concentration of the antibody was measured in m-CD24-expressing T47D/vector and MDA468/shIRIS cells (Figure 5A and 5D, respectively). In contrast, the same treatment had no effect on the c-CD24-expressing T47D/IRIS and MDA468/shcontrol cells (Figure 5B and 5C, respectively), suggesting that the intrinsic sensitivity to anti-CD24 cross-linking antibody in parental T47D could be acquired by IRIS silencing in MDA468. These effects were even more pronounced at 96h and 120h (Figure 5A-5D).

### Further evidence for IRISOE driving the TNBC/TIC phenotype

During our preliminary studies, we noticed that the duration of incubating MCF7 cells (we tested several clones from several labs and obtained the same result) with FITC-CD44 and PE-CD24 affects the FACS profile. Longer duration (1hr) leads to a profile similar to that shown in Figure 3E with no TIC/CD44^+^CD24^-^ cells observed. However, shorter duration (20mins) produced the profile shown in Figure 5E with ∼20% of the cells showing the TIC/CD44^+^CD24^-^ profile (circle A, Figure 5E). Reasons and implications for this observation are still unclear.

We took advantage of this observation and FACS sorted MCF7 labeled for only 20mins into TIC/CD44^+^CD24^-^ cells (circle A, Figure 5E) and non-TIC/CD44^-^CD24^+^ cells (circle B, Figure 5E). However, because the two populations are near each other, we ensured accuracy by only collecting cells at the periphery of the circles and not the intersection. We also FACS sorted T47D cells into non-TIC/CD44^-^CD24^+^ cells and TIC-like/CD44^-/low^CD24^-^ cells (see Figure 3G). *RNAs* and proteins from each fraction were isolated. As expected, *IRIS mRNA* was detected in TIC/CD44^+^CD24^-^ MCF7 and TIC-like/CD44^-/low^CD24^-^ T47D cells only (compare lanes 2 and 4 to 1 and 3, respectively, Figure 5F). Using real-time QRT/PCR, we showed an increase in stemness-enforcers (*Oct4*, *Sox2* and *Nanog*), basal biomarkers (*EGFR*, *CK5*, *CK17* and *CDH3*), and EMT-inducers (*Twist*, *slug*, *fibronectin*, *vimentin* and *CDH2*) *mRNAs* expression in the TIC compared to the non-TIC MCF7 (black bars in Figure 5G), and T47D (red bars in Figure 5G) cells. This was confirmed on the protein level as well, where sorted TICs and not non-TICs from both cell lines showed higher/exclusive IRIS, stemness-enforcers (Figure 5H), basal-biomarkers (Figure 5I), and EMT-inducers (Figure 5J) proteins expression. Taken together, these data strongly support the view that IRISOE is associated with high expression of basal-biomarkers, EMT-inducers and stemness-enforcers in TICs isolated from any breast cancer subtype.

Mammosphere formation assay (MSF) is a highly accurate *in vitro* measure of stemness in breast cancer cells. We plated equal numbers of FACS sorted TIC/CD44^+^CD24^-^ MCF7, non-TIC/CD44^-^CD24^+^ MCF7 cells, TIC-like/CD44^-/low^CD24^-^ T47D, and non-TIC/CD44^-^/CD24^+^ T47D cells in low binding wells for 14 days (we find that longer duration is required for luminal A cells to form MSFs), at which time the number and diameter of MSFs formed were assessed. Compared to non-TIC/CD44^-^/CD24^+^ isolated from both cell lines (that only formed very small MSFs, Supplementary Figure 4A and 4C), TIC/CD44^+^CD24^-^ MCF7 cells and TIC-like/CD44^-/low^ CD24^-^ T47D cells formed many MSFs (left, Figure 5K, and Supplementary Figure 4B and 4D) that showed larger size (right, Figure 5K, and Supplementary Figure 4B and 4D), supporting our conclusion that IRISOE cells isolated from any breast cancer subtype are indeed TICs.

Finally, we plated equal number of unsorted MCF7 cells (i.e. IRIS^low^, Figure 5L, left), non-TIC/CD44^-^CD24^+^ (i.e. IRIS^-^, Figure 5L, left) or TIC/CD44^+^CD24^-^ (i.e. IRISOE, Figure 5L, left) sorted MCF7 cells in low binding wells. After 7 days when MSFs were visible, some TIC/CD44^+^CD24^-^ cultures were transfected with siLuc or siIRIS (Figure 5L, right) alone or in combination with 5μg/ml anti-CD24 cross-linking antibody and incubated for an additional 7 days. Again, unlike the unsorted MCF7 cells that showed low MSF capacity (Figure 5N), and the non-TIC/CD44^-^CD24^+^ sorted MCF7 cells that formed low/no MSFs (Figure 5M), the TIC/CD44^+^CD24^-^MCF7 cells formed many large MSFs (Figure 5O). As expected, treatment of TIC/CD44^+^CD24^-^ MCF7 MSFs with the anti-CD24 cross-linking antibody alone had no effect (they are CD24^-^, compare Figure 5P-5O). In contrast, IRIS silencing alone through promoting cell death (arrows in Figure 5Q) partially reduced the size of MSFs (compare Figure 5Q-5O). Treating IRIS silenced MSFs with the anti-CD24 cross-linking antibody completely abolished these MSFs (compare Figure 5R-5O). The schematic shown in Figure 5S, summarizes our hypothesis based on data presented. We propose that in TNBC/TICs through enhancing expression/activity of the signaling pathway EGFR→c-Src→cortactin, IRISOE promotes their invasive (promoting invadopodia) and TIC (promoting m-CD24 internalization, black arrows in Figure 5S) phenotypes. IRIS silencing (si/shRNA) or inactivation (inhibitory peptide) suppresses expression/activity of this signaling pathway leading to loss of the invasive ability and by re-surfacing of CD24 to loss of the TIC phenotypes and enhanced sensitization to an anti-CD24 cross-linking therapy in TNBC/TICs (red arrows in Figure 5S).

### IRISOE promotes formation of breast cancers composed of TICs

**Figure 6:**
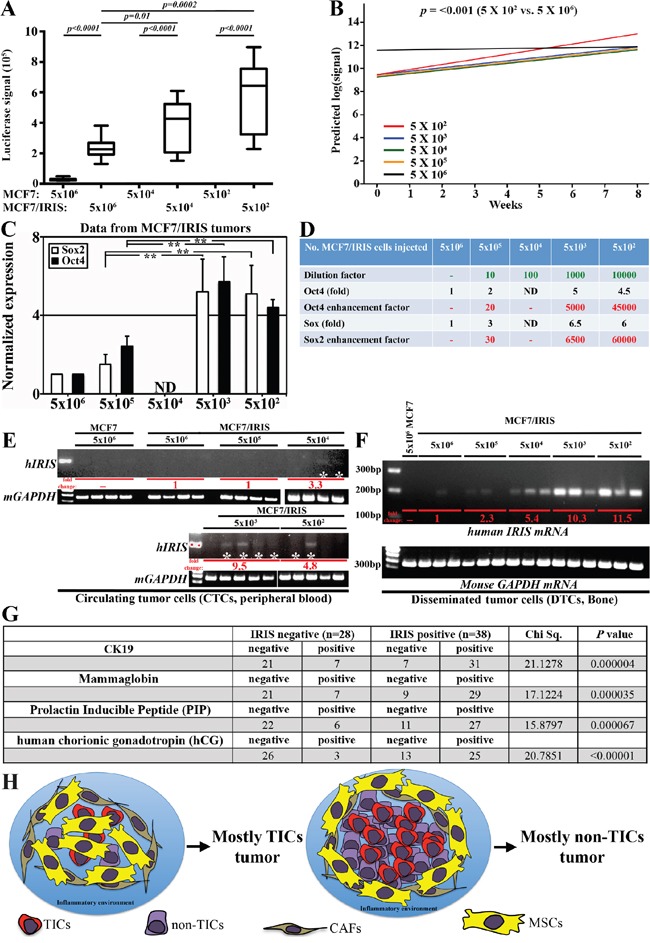
IRISOE promotes formation of TIC tumors, their dissemination and metastasis A. Luciferase signals obtained in tumors developed using different concentration of parental or IRISOE MCF7 at week 8 post cells injection. B. Slopes of curves generated for luciferase signals for tumors developed using MCF7/IRISOE cells originally injected at the indicted numbers plotted against time (weeks). C. Real time QRT/PCR analysis for human Sox2 and Oct4 mRNAs expression in tumors developed using MCF7/IRISOE cells originally injected at the indicated numbers at week 8. D. Assessment of the stemness enhancement factor in tumors developed using MCF7/IRISOE originally injected at the indicated numbers extrapolated from the data presented in (C). RT/PCR analysis for human IRIS mRNA (upper) or mouse GAPDH mRNA (loading control, lower) in PB E. or BM F. samples from mice originally injected with the indicated cell lines at the indicated numbers. G. Association between IRIS mRNA expression and the expression of the dissemination biomarkers; CK19, MGA, PIP, hGC mRNAs in a cohort of metastatic breast cancer patients (n=66, from Egyptian NCI) conducted using real-time QRT/PCR. H. Schematic presentation of our overall hypothesis that bidirectional interactions with the microenvironment enhance IRISOE/TNBC/TIC early lesion formation, aggressiveness, and eventually dissemination and metastasis.

Breast cancer formation using low number of cells is an accurate measure of the presence of TICs within the injected cells. Therefore, we injected 5×10^6^, 5×10^5^, 5×10^4^ 5×10^3^ or 5×10^2^ of parental or IRISOE MCF7 cells in mammary fat pads of female Nu/Nu mice (n=5 per cell line/concentration). Tumor formation was followed up for 8 weeks using caliper and *in vivo* luciferase imaging. None of the mice injected, regardless of the number of the parental MCF7 cells showed any luciferase signal throughout the 8 weeks, indicating no tumor formation (Figure 6A). In contrast, all the mice injected with MCF7/IRISOE cells showed luciferase signal indicating tumor formation even in the absence of exogenous oestrogen (E2, Figure 6A). Thus, IRISOE enhances MCF7 cells ability to form E2-independent tumors.

Surprisingly, tumors developed by injecting lower number of MCF7/IRISOE cells (e.g., 5×10^2^) were larger in their luciferase signal than those developed by injecting higher number of cells (e.g., 5×10^6^, Figure 6A). To clearly illustrate this point the mean luciferase signal in each group was first converted into log and the slope for these curves were plotted against time. Slope of the signal obtained from tumors developed by injecting 500 MCF7/IRISOE cells was associated with the highest rate of increments compared to those obtained by injecting 5×10^6^ MCF7/IRISOE cells (0.463 vs. 0.026 *p<0.001*, compare red to black line in Figure 6B). We propose that since IRISOE converts non-TIC/CD44^-^/CD24^+^ MCF7 cells into TIC/CD44^+^/CD24^-^ MCF7 cells (compare Figure 3F-3E) injecting lower number of cells allows IRISOE/TIC cells to form stronger interactions with the stroma leading to exponential growth of these TIC tumors. It is possible that such interactions with the stroma are less likely when injecting higher number of the same cell line (see discussion).

To establish this further, total *RNAs* were isolated from all tumors and were subjected to real time QRT/PCR analysis using primers that amplify human not mouse *Sox2, Oct4* or *GAPDH mRNAs*. The data for *Sox2* or *Oct4 mRNAs* in each sample were first normalized to the level of *GAPDH mRNA* in each sample and then to the level found in tumors developed following injection of 5 × 10^6^ MCF7/IRISOE cells (i.e. taken as 1, Figure 6C). Two fold increase in *Oct4 mRNA* level and 3 fold increase in *Sox2 mRNA* level in tumors developed following injection of 5 × 10^5^ MCF7/IROSOE cells, 5 fold increase in *Oct4 mRNA* level and 6.5 fold increase in *Sox2 mRNA* level in tumors developed following injection of 5000 MCF7/IROSOE cells, and 4.5 fold increase in *Oct4 mRNA* level and 6 fold increase in *Sox2 mRNA* level in tumors developed following injection of 500 MCF7/IRIS cells was measured (Figure 6C). Using the formula “enhancement factor (i.e. IRISOE/TICs expansion) = dilution factor X marker fold increase” we estimated that compared to TICs in tumors developed by injecting 5 × 10^6^ MCF7/IRISOE, the TICs expanded 20-30 times when 5 × 10^5^ MCF7/IRISOE cells were injected, 5000-6500 times when 5000 MCF7/IRISOE cells were injected, and of 45000-60000 times when 500 cells MCF7/IRISOE were injected (Figure 6D), re-enforcing the above conclusion that within a low number of IRISOE/TIC/CD44^+^/CD24^-^ MCF7 cells stronger interactions with the stroma lead to formation of TIC tumors (see discussion).

### IRISOE induces breast cancer cells early dissemination

The data presented above also imply increased aggressiveness in tumors formed by injecting lower number of MCF7/IRISOE cells. Dissemination and metastasis are accurate measures of such aggressiveness. The premise of the next experiments is that lower number of cells originally injected could mimic early lesions in TNBC patients. We collected peripheral blood (PB) and bone marrows (BM) from all the mice described above, removed red blood cells from PBs and isolated total RNAs from all PB and BM samples. To study circulating tumor cells (CTCs), PB samples were analyzed using RT/PCR with pair of primers that only amplify intron 11 domain of human *IRIS mRNA* (intron 11 in mouse and human *IRIS mRNAs* are completely divergent). As expected, no amplifications were detected in PB of mice injected with parental MCF7 even at the highest number of cells injected (5 × 10^6^, Figure 6E). The very faint signal obtained in PBs of mice originally injected with 5 × 10^6^ MCF7/IRISOE was taken as 1 (red letters, Figure 6E). No increase in level of CTCs (i.e. human *IRIS mRNAs* PCR amplification signal) in PB of mice originally injected with 5 × 10^5^ MCF7/IRISOE was detected (red letter, Figure 6E). In contrast, a 3.3, 9.5 and 4.8fold increase in CTCs (i.e. human *IRIS mRNAs* PCR amplification signal) in PB of mice originally injected with 5 × 10^4^, 5 × 10^3^, and 5 × 10^2^ MCF7/IRISOE, respectively were measured (red letters, Figure 6E).

To study Disseminated tumor cells (DTCs), BM samples were analyzed using RT/PCR with human *IRIS-intron 11* primers on *RNAs* isolated from BM from all the mice described above. Again no human *IRIS mRNA* was amplified from BM of mice injected with any even 5×10^6^ MCF7 cells (Figure 6F). We again considered RT/PCR signal obtained from BM of mice injected with 5 × 10^6^ MCF7/IRISOE as 1 (red letter, Figure 6F). Accordingly, 2.3fold, 5.4fold, 10.3fold, and 11.5fold increase in DTCs (i.e. human *IRIS mRNAs* PCR amplification signal) were measured in BM of mice injected with 5 × 10^5^, 5 × 10^4^, 5 × 10^3^, and 5 × 10^2^ MCF7/IRISOE cells, respectively (red letter, Figure 6F). Taken together, these data raise the interesting possibility that IRISOE/TNBC/TICs disseminate from early lesions (mimicked by low number of cells injected), and metastasize.

To support this hypothesis further, the metastatic breast cancer cohort (n=66) described above was also evaluated for the expression of several biomarkers associated with breast cancer dissemination/metastasis, namely CK19 [50], mammaglobin-A (MGA) [51], prolactin inducible peptide (PIP) [52], and human chorionic gonadotrophin (hCG) [53]. Quantitative real-time RT/PCR showed that among the IRIS-negative tumors (n=28), 75% (21/28) were negative, and 25% (7/28) were positive, while among the IRIS-positive tumors (n=38), 18% (7/38) were negative, and 89% (31/38) were positive for CK19 (Chi Sq.=21.1278, *p=0.000004*, Figure 6G). Among the IRIS-negative tumors, 75% (21/28) were negative, and 25% (7/28) were positive, while among the IRIS-positive tumors, 24% (9/38) were negative, and 76% (29/38) were positive for MGA (Chi Sq.=17.1224, *p=0.000035*, Figure 6G). Among the IRIS-negative tumors, 79% (22/28) were negative, and 21% (6/28) were positive, while among the IRIS-positive tumors, 29% (11/38) were negative, and 81% (27/38) were positive for PIP (Chi Sq.=15.8797, *p=0.000067*, Figure 6G). Finally, among the IRIS-negative tumors, 89% (26/28) were negative, and 11% (3/28) were positive, while among the IRIS-positive tumors, 34% (13/38) were negative and 66% (25/38) were positive for hCG (Chi Sq.=20.7851, *p<0.00001*, Figure 6G).

Finally, analysis of the TNBC cohort (n=72) described above showed that the metastatic status between IRIS-negative/TNBC tumors (n=13) and IRIS-positive/TNBC tumors (n=59) was not statistically significant (*p=0.570703*, Supplementary Figure 5). However, in this cohort 31% (22/72) patients showed lymph-node (LN) metastasis and 8% (6/72) patients showed distant metastasis (DM). From the 22 patients that showed LN metastasis, 18% (4/22) were IRIS-negative, while 82% (18/22) were IRIS-positive, and all the patients that showed DM were IRIS-positive (Supplementary Figure 5). Taken together, these data strongly implicate that in pre-clinical mouse models and TNBC patients IRISOE/TNBC/TIC cells disseminate more efficiently from smaller tumors/early lesions, respectively.

### Inhibiting IRIS promotes regression of TIC tumors

**Figure 7:**
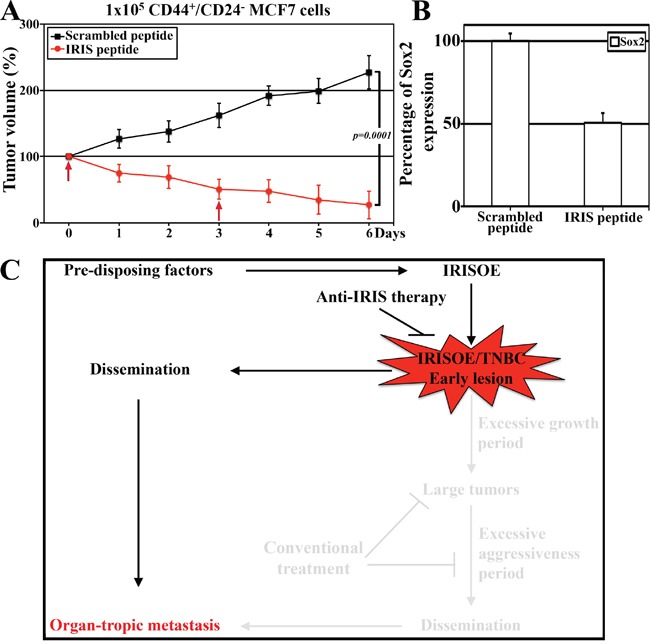
The efficacy of IRIS inhibitory peptide against TIC tumor cells, in vivo A. The growth of established TIC/CD44+CD- MCF7 tumors grown in NSG mice following scrambled or IRIS-inhibitory intratumoral injection at the indicated times (arrows). B. Real-time QRT/PCR analysis for the expression of human Sox2 mRNA in the tumors found in the mice at day 7 (in A) post-injection of scrambled or IRIS-inhibitory peptide. C. Schematic presentation of our data combined and the hypothesis that finding and targeting the early lesions could be much more beneficial for IRISOE/TNBC patients.

Finally, to establish the efficacy of targeting IRISOE to prevent formation of TNBC/TIC tumors and their early dissemination, we injected five hundred thousand CD44^+^/CD24^-^ sorted MCF7 cells into the mammary fat pads of 10 female NSG mice. When tumors were established (∼0.1 cm^3^), mice were randomly divided into 2 groups that were injected intratumorally with scrambled or IRIS-inhibitory peptide (10mg/kg/day) on day 0 and 3 (arrows, Figure 7A). Tumor growth was measured by caliper daily and on day 7 post-treatments, all tumors were taken and RNAs were prepared. First, while the growth of these established tumors treated with scrambled peptide continued to increase to reach ≥200% one week later, only ∼20% of the original tumor volume remained on day 7 following IRIS-inhibitory peptide injection (compare red to black line, Figure 7A). Second, real-time QRT/PCR analysis using primer set that amplifies human mRNAs showed that after normalization to *GAPDH mRNA* level in each sample, compared to *Sox2 mRNA* level in scrambled peptide injected tumors, the expression level of *Sox2 mRNA* was decreased by 50% in IRIS-inhibitory peptide treated tumors (Figure 7B), suggesting that the regression measured is due to IRIS inactivation-induced decrease in the pool of TICs within the tumor. Taken together, these data support the hypothesis that IRIS inhibition could be beneficial in inhibiting the formation and dissemination of TNBC/TIC tumors in patients (Figure 7C).

## DISCUSSION

In this study we demonstrate that IRISOE suppresses BRCA1 protein expression, while enhances basal-biomarkers, EMT-inducers, stemness-enforcers expression, and the TIC phenotype all are known TNBC cells associated events. With regard to the effect on BRCA1 expression, we recently showed that IROSE ERα function in ERα/BRCA1-expressing breast cancer cells (e.g., MCF7) [40]. Since *BRCA1* transcription is indirectly activated by E2/ERα [54], suppressing ERα could negatively impact *BRCA1* transcription. *ESR1* transcription is directly activated by BRCA1 [55], which suggest that IRISOE could fuel this negative feedback events culminating on suppression of BRCA1 expression. Additionally, recent studies showed Oct4, Nanog, Slug, or ALDH1 overexpression in MCF-7 cells could reduce ERα expression [56, 57], thus because IRISOE upregulates the expression of all these factors, this could be yet another mechanism to explain IRISOE negative effect on BRCA1 expression in TNBC cells.

The role of m-CD24 in cancer progression seems complicated with some studies show promotion role [58, 59], while others associate c-CD24 instead with cancer progression [7, 60–62]. The current studies IRISOE-induced accumulation of c-CD24 association with increased aggressiveness in TNBCs in animal models [38] and humans (this study) are consistent with previous studies. For example, in one study strong c-CD24 expression was correlated with shortened survival in colorectal cancer patients even without distant metastases [21, 22, 63, 64]. In another, c/m-CD24 expression was shown to be specific a marker for tamoxifen-resistant breast cancer cases [25]. A third study showed that CD44^+^CD24^-/low^ cells were concentrated in breast cancers invasive protrusions [26].

Loss of cell polarity during EMT changes apical m-CD24 in normal and benign lesions into c-CD24 pattern in high-grade tumors [8]. Overexpression of EMT inducers, e.g., Slug increases CD44^+^/CD24^-^ cell number [65]. Thus, in addition to promoting CD24 internalization (this study), IRISOE by promoting EMT-inducers expression [36] could enhance breast cancer TICs formation. EGFR overexpression in the majority of TNBCs [5, 66], the close association between EGFR overexpression and activated p-c-Src^Y416^ membrane localization in TNBCs [67, 68], cortactin involvement in promoting stem-like phenotype as well as endocytosis in many cancers [30, 33, 34, 47, 69], and the fact that IRIS is overexpressed in 88% [38] EGFR is overexpressed in 75% [1, 70], vimentin is overexpressed in 88% [1, 6] and CD44 is overexpressed in 77% [71] of TNBCs, all support the internalization hypothesis as the major mechanism involved in IRISOE-induced CD24 cytoplasmic confinement in TNBC/TICs.

CD24 cross-linking antibody induced apoptosis in human and mouse m-CD24-expressing B cells [48], reduced growth and migration in human m-CD24-expressing colorectal and breast cancer cell lines [49, 63], and now in IRISOE/TNBC/TICs (this study). Because cell death induced by the CD24 cross-linking antibody was shown previously to be through mitochondrial regulation (reactive oxygen species generation and/or Bcl-2 inhibition), we currently investigating whether in IRSOE/TNBC/TICs, mitochondrial regulation could be another possible combinatorial therapy in addition to IRIS inhibitor + anti-CD24 blocking antibody to benefit breast cancer patients with localized BRCA1-low/IRISOE/TNBCs.

An important unanswered question is highlighted from the data presented: how IRISOE promotes stemness-enforcers, EMT-inducers, and basal-biomarkers expression in mammary epithelial cells? It is possible that IRISOE by specifically upregulating Sox2 expression (see Figure 2F and 2G, and 5G and 5H, 6C, and 7D) enhances Oct4 and Nanog expression as previously reported [72, 73], enhances expression of the EMT-inducers (e.g., Twist, Slug, or Snail) also as previously reported [74, 75]. Alternatively, IRISOE through enhancing expression of basal-biomarkers, such as, EGFR and its ligand EGF [36] could initiate a signal transduction that culminates on enhancing expression of EMT-inducers [76], stemness-enforcers [77], and basal-biomarkers expression. Yet, a more intriguing possibility is that by negatively affecting p53 [78] and/or BRCA1 (see Figure 1A-1D) [38], IRISOE could upregulate expression of EMT-inducers, stemness-enforcers, and basal-biomarkers [79].

IRISOE converted 40% of MCF7 cells from non-TIC into TICs (compare Figure 3F-3E), suggesting that within 5 × 10^6^ MCF7/IRISOE cells injected there are 2 × 10^6^ TICs, while within the 500 cells injected there are 200 TICs. As schematically presented in Figure 6H, TICs within the lower number of cells injected are in close proximity to microenvironment elements, such as, tumor activated fibroblasts (CAFs), mesenchymal stem cells (MSCs), the inflammatory and hypoxic environment, as well as other TICs (Figure 6H, left). Bi-directional interactions with CAFs, MSCs, etc. could increase these TICs aggressiveness, which could be exacerbated even further by the inflammatory and hypoxic environment. Bi-directional interactions with other TICs could also positively impact cells’ aggressiveness culminating on the expansion TICs. Bi-directional interactions between TICs and non-TICs could induce de-differentiation of non-TICs and converts them into TICs and increase the starting pool of aggressive TICs [80, 81]. These interactions are less likely to occur when larger number of cells are injected, which could lead to the depletion of the pool of TICs, and negatively impact their aggressiveness (Figure 6H, right). We propose that an early TNBC lesion develop in pre-disposed patients when microenvironment event(s) upregulates IRIS expression in a selected mammary cells (cell of origin of future TNBC/TIC tumor), which through bi-directional interactions with other elements of microenvironment develop into IRISOE/TNBC/TICs tumors (lighted part, Figure 7C). Thus therapeutic interventions at later stage as it in current clinical settings would be less beneficial especially in those patients (shaded part, Figure 7C). Moreover, dissemination is thought to occur as a late step from large tumors (shaded part, Figure 7C). Our data consider a different model for dissemination from aggressive IRISOE/TNBCs. We propose that IRISOE/TNBC/TICs can disseminate from early lesion and metastasize (lighted part, Figure 7C). Clinical studies that show TNBCs as oppose to ER^+^ cancers relapse within 5 years after surgery [87–89] support our hypothesis. If true, identifying the early IRISOE/TNBC/TIC lesions and target them at early stage with an IRIS-specific therapy might eliminate dissemination/metastasis of TNBCs at an early stage (lighted part, Figure 7C). Finally, the failure of MCF7 cells to form tumors could be due to their differentiation [82, 83]. Differentiated cells are known to be immunogenic, while progenitors are non-immunogenic [84]. If true, this suggests that IRISOE promotes tumor formation by promoting cells de-differentiate-induced immune evasion, *in vivo* [85, 86].

The fact that TNBCs form visceral metastasis and IRISOE/MCF7 formed bone metastasis, could be explained by: if we waited for >8 weeks, we would have measured other organ metastasis. Alternatively, this could be perhaps the nature of the luminal A/MCF7 cells, and if we re-inject the MCF7/IRISOE that homed to bone, we could produce genuine TNBC metastatic pattern.

## MATERIALS AND METHODS

### Cell culture

All breast cancer cell lines were obtained from American Type Culture Collection (ATCC) and maintained as previously described [31, 34]. HME cells were maintained as previously described [31]. The doxycycline inducible BRCA1-IRIS overexpressing cell lines were generated and maintained as described earlier [35].

### Antibodies

All antibodies used in this study are listed in Supplementary Table 1.

### Western blot analysis

For whole cell proteins extraction, samples were sonicated using a probe sonicator. Nuclear and cytoplasmic proteins preparation was as follows. Washed cells were re-suspend in buffer A (containing: 10mM HEPES, 10mM KCl, 0.5mM DTT + 1% v:v NP-40 + protease inhibitors) and incubate at 4°C for 10 min with rocking, followed by centrifugation at high speed for 2 min at 4°C, and collecting the supernatant (cytoplasmic proteins). Pellet was re-suspended in buffer B (20mM HEPES, 500mM KCl, 1.5mM MgCL_2_, 0.5mM DTT, 20% Glycerol + protease inhibitors) and incubated at 4°C for 15 min with rocking, followed by centrifuge at high speed 5-10mins at 4°C, and collecting the supernatant (nuclear proteins). For membrane proteins isolation abcam kit (ab65400) was used. Homogenized cells were suspended in homognizer buffer in ice-cold Dounce homogenizer on ice for 30-50 times, sonicated, and centrifuged at 700 × g, 10 min at 4°C. Discard pellet (containing nuclei and debris) and centrifuge the supernatant at 10,000 x g for 30 min at 4°C. Collect the supernatant (the cytosol fraction), and the pellet containing total cellular membrane proteins is purified according to manufacturer procedures. In some cases cytoplasmic and membrane proteins were mixed. Protein concentrations were estimated using Pierce™ BCA protein assay kit (Thermo Scientific, Waltham, MA, USA). Cell lysates were denatured in NuPAGE LDS sample buffer (Thermo Scientific) and were resolved on NuPAGE gels (Thermo Scientific) and electro-transferred to PVDF membrane. Membrane was blocked with 5% dry milk, washed and subsequently incubated with primary antibody overnight at 4°C. Blots were then incubated with HRP-conjugated secondary antibody for 1 h at RT, washed and developed using Western lightening Plus-ECL as a substrate. Tubulin and actin were used as an internal loading control. Each western blot was performed at least 2 times with identical results.

### Immunofluorescence staining

Performed according to protocol described in [32]. Each staining was performed at least 2 times with identical results.

### Human samples analysis

The study involved a cohort (n=326) of breast cancers that contained a sub-cohort of TNBCs (n=72) from the Hawaiian SEER collection. The study also involved two additional cohorts the first recently diagnosed patients with locally advanced breast cancers (n=49), and another recently diagnosed patients with metastatic breast cancer diagnosed patients (n=66), who were diagnosed and treated at the National Cancer Institute (NCI), Cairo University, (Cairo, Egypt) between September 2009 and October 2012 [90, 91]. For the Egyptian cohorts, normal breast tissue samples (n=20) obtained from females undergoing reduction mammoplasty were included in the study as a control group (all three groups were matched for age). Tumors were graded according to the most recent WHO classification of breast tumors and staged according to the American Joint Committee on Cancer's Staging Manual, 7th edition [92, 93]. Written informed consent was obtained from all participants prior to enrollment in the study. The Institutional Review Board (IRB) of the NCI, Cairo, Egypt approved the protocol, in accordance to the 2011 Declaration of Helsinki. Patients enrolled in the study were ≥18 years old, had an Eastern Cooperative Oncology Group (ECOG) Adequate performance: ≤2 [94], and exhibited adequate hematological parameters (WBC count, ≥3.0×10^9^/l; ANC, ≥1.5×10^9^/l; platelet count, ≥100×10^9^/l; hemoglobin level, ≥9 g/l), liver function (serum bilirubin, <1.5×ULN; ALT and AST levels, <3 times normal values), and kidney function (plasma creatinine level, <1.5 times normal value) function. None of the patients were pregnant or breast-feeding, had an active second malignancy, or were involved in another clinical trial were excluded from the study. The median follow-up period was 33 months.

### Immunohistochemistry (IHC)

IHC analysis was performed on 4-5μm thick paraffin-embedded sections of tumor tissue excised from breast cancer patients or IRISOE orthotopic mammary tumor generated in SCID, Nu/Nu, or NSG mice. Briefly, sections were deparaffinized, rehydrated, and washed in phosphate-buffered saline (PBS). Antigen retrieval for IRIS staining was performed by incubating the slides in pepsin (10μM) for 20min at 37°C. Antigen retrieval for all other antigens was performed by boiling the slides in citrate buffer (pH 6.0) for 10min in the microwave. Slides were then cooled to RT and washed 3 times with PBS for 15 min each. Slides were incubated in 3% hydrogen peroxide (H_2_O_2_) for 10min to block endogenous peroxidase activity unless fluorescence analyses were performed. After washing, slides were blocked with 10% normal goat serum for 1h at RT and subsequently probed with primary antibodies overnight at 4°C. After three PBS washes slides were incubated with Horseradish peroxidase (HRP), or Alexa Fluor (488 or 647) conjugated secondary antibody for 1h at RT. After washing, slides were stained with HRP-conjugated secondary antibody were developed with Vector DAB substrate kit (Vector laboratories, Burlingame, CA, USA) and counterstained with Meyer's hematoxylin (Thermo Scientific) for 2 min. Alternatively, slides stained with Alexa Fluor conjugated secondary antibody were counterstained and mounted with VECTASHIELD mounting medium for fluorescence with DAPI (Vector laboratory Inc, Burlingame, CA, USA) observed under microscope.

### Fluorescence-activated cell sorting (FACS)

One million cells were stained with FITC-CD44 and PE-CD24 antibodies for 30min. For ALDH1 activity, cells were stained using the ALDEFLUOR™ Kit (STEMCELL Technologies). Both analyzed on Gallios Flow Cytometer (Beckman Coulter). For sorting Moflo XDP cell sorter (Beckman Coulter) was used. Data was analyzed with Kaluza Flow Cytometry Analysis Software v 1.2. Each FACS was performed ≥2 times with identical results.

### Mammosphere formation assay

One thousand cells were plated in ultra-low binding dishes (Corning) in recommended medium that was changed every 3^rd^ day for up to 2 weeks and mammospheres were counted and photographed. Each experiment was done in triplicate, three separate times.

### RT/PCR and real-time quantitative RT/PCR

One hundred ng of total RNA was processed for RT/PCR using SuperScript One-Step RT-PCR with Platinum Taq (Invitrogen) kit and for quantitative RT-PCR (qRT-PCR) using iScript™ One-Step RT-PCR kit with SYBR Green (Bio-Rad) using primers listed in Supplementary Table 2. *GAPDH mRNA* was used as internal control and all done in triplicates performed 3 separate times.

For human samples RNA was extracted from representative tumors and normal breast tissue samples using the RNAeasy Mini Kit (Qiagen, Milan, Italy), and retro-transcribed using iScriptTMcDNA Synthesis Kit (Bio-Rad, Milano, Italy) according to the manufacturer's instructions. Relative mRNA levels of the studied markers were measured in triplicates using the Syber Green technique according to manufacturer's protocols (Applied Biosystems, Inc., Foster City, CA, USA), with the following primer sequences for the BRCA1-IRIS (Forward: GTCTGAGTGACAAGGAATTGGTTT; Reverse: TTAACTATACTTGGAAATTTGTAAAATGTG) normalized with β-actin as a house-keeping gene (Forward: ACAGAGCCTCGCCTTTGC; Reverse: GCGGCGATATCATCATCC) and expressed in relation to calibrator sample. The qPCR reactions were performed in a final volume of 25μl. The mean Ct was calculated for each sample and used to determine the ΔCt for this sample as follows: ΔCT = Ct for the gene of interest - Ct of the internal control gene (β-actin). Then the ΔΔCT was calculated as follows: ΔΔCT = [(Ct for the gene of interest - Ct of the internal control gene, β-actin) for sample A - (Ct for the gene of interest - Ct of the internal control gene (β-actin) for sample B], where sample B is the calibrator. For the statistical analysis, the ΔΔCT and not the raw Ct data were used [95].

### Orthotopic mammary tumor model and *in vivo* peptide treatment

All animal experiments were approved by ‘Institutional Animal Care and Use Committee’ (IACUC) of University of Mississippi Medical Center. Orthotopic mammary tumors were established by injecting cells in 2^nd^ left and/or 4^th^ right mammary fat pads of 6-8 weeks old female Nu/Nu mice. Tumors were measured with digital caliper every 3^rd^ day and luciferase imaging weekly on IVIS SPECTRUM (PerkinElmer), and data analyzed using LivingImage v 4.3.1 software. Tumors were resected and digested with collagenase-A/trypsin for quantitative RT-PCR analysis. Bone marrow was flushed and RNA was isolated, whereas peripheral blood was immediately treated with RBC lysis buffer (BioLegend) to remove red blood cells and remaining cells were used to isolate total RNA. Additionally, 6 weeks old NSG (NOD.Cg-Prkdc^scid^ il2rg^tm1Wjl^/SzJ) female mice were injected with 1×10^5^ CD44^+^/CD24^-^ sorted MCF7 cells. When tumors reached ∼100mm^3^, mice were divided into two groups that were injected with scrambled or IRIS-inhibitory peptide (10mg/kg/day, intratumorally) on day 0 and 3. Tumors excised on day 7 were used to generate single cell suspension or to isolate total RNA for quantitative RT/PCR.

### Statistical analysis

SPSS, version 20.0 (IBM SPSS, Armonk, NY, USA) was used for statistical analysis. Data were expressed as the mean rank or mean ± standard deviation for continuous variables. We examined the association between *IRIS mRNA* expression with other clinico-pathological variables, and the significance of studied markers using Chi Square (χ^2^) test. All P-values are two-tailed and P<0.05 was considered to indicate a statistically significant difference. Comparisons of biomarkers between different groups were performed using the Mann-Whitney U test or Kruskal Wallis one-way analysis of variance. Metastasis association was analyzed using Kaplan-Meier curve and as compared using the log-rank test and Cox regression analysis to adjust for other prognostic indicators.

## SUPPLEMENTARY FIGURES AND TABLES


