# Supplementary material for: BRCA1-IRIS overexpression promotes and maintains the tumor initiating phenotype: implications for triple negative breast cancer early lesions
Source: Oncotarget. 2016 Dec 29;8(6):10114–35. doi: 10.18632/oncotarget.14357 (PMC5354646; doi:10.18632/oncotarget.14357)
Supplement: Supplementary file 1 [file oncotarget-08-10114-s001.pdf]

BRCA1-IRIS overexpression promotes and maintains the tumor initiating phenotype: implications for triple negative breast cancer early lesions

SUPPLEMENTARY FIGURES AND TABLES

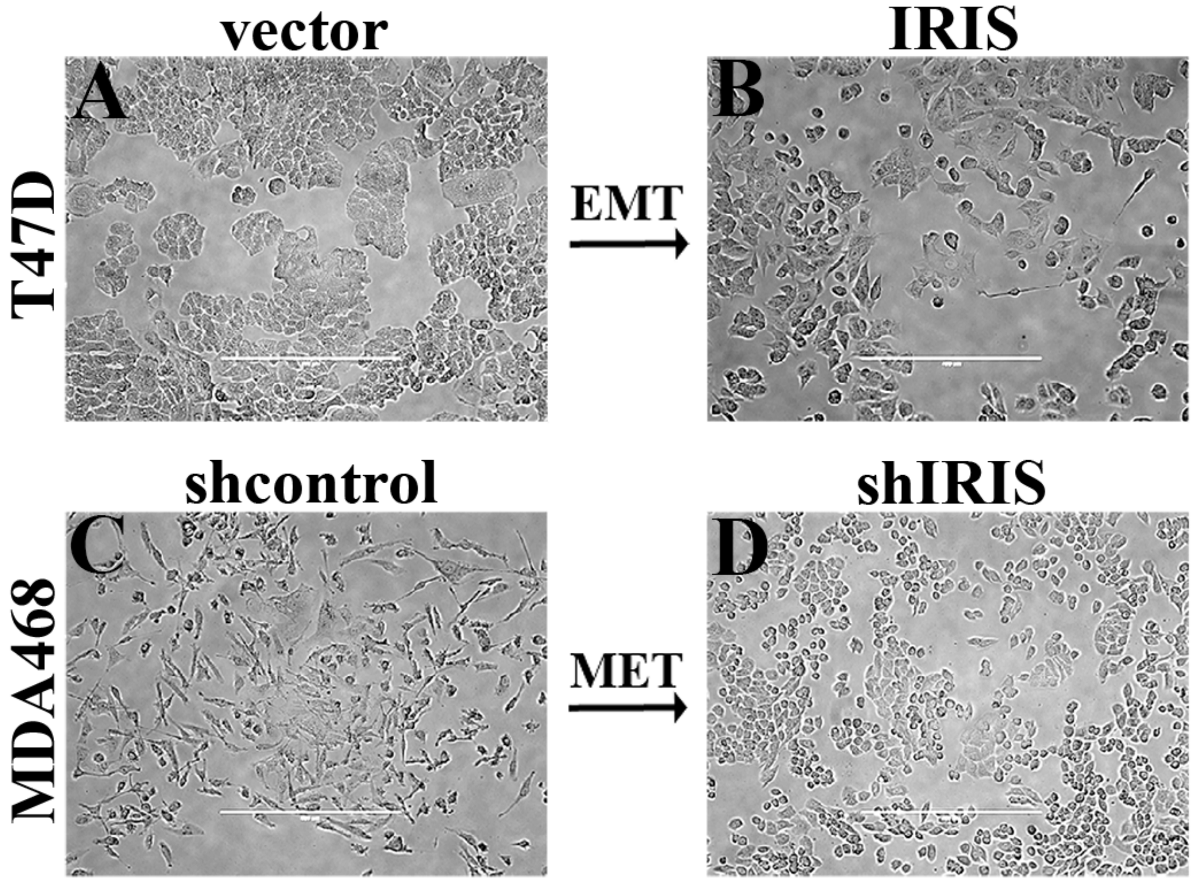

Supplementary Figure 1 (related to Figure 2): A and **B.** IRIS overexpression induces EMT morphology in the luminal cell line (T47D). **C** and **D.** IRIS silencing induces MET in the TNBC cell line (MDA468).

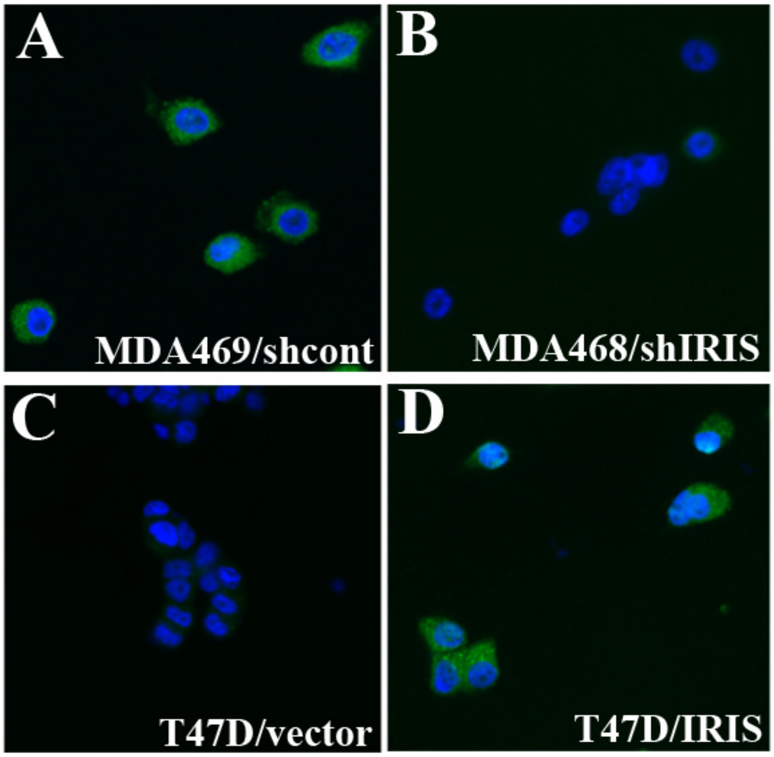

Supplementary Figure 2 (related to Figure 3): ALDH1 expression in MDA468 cells expressing shcontrol **A.**, shIRIS **B.**, or in T47D expressing vector **C.** or IRIS cDNA **D.**

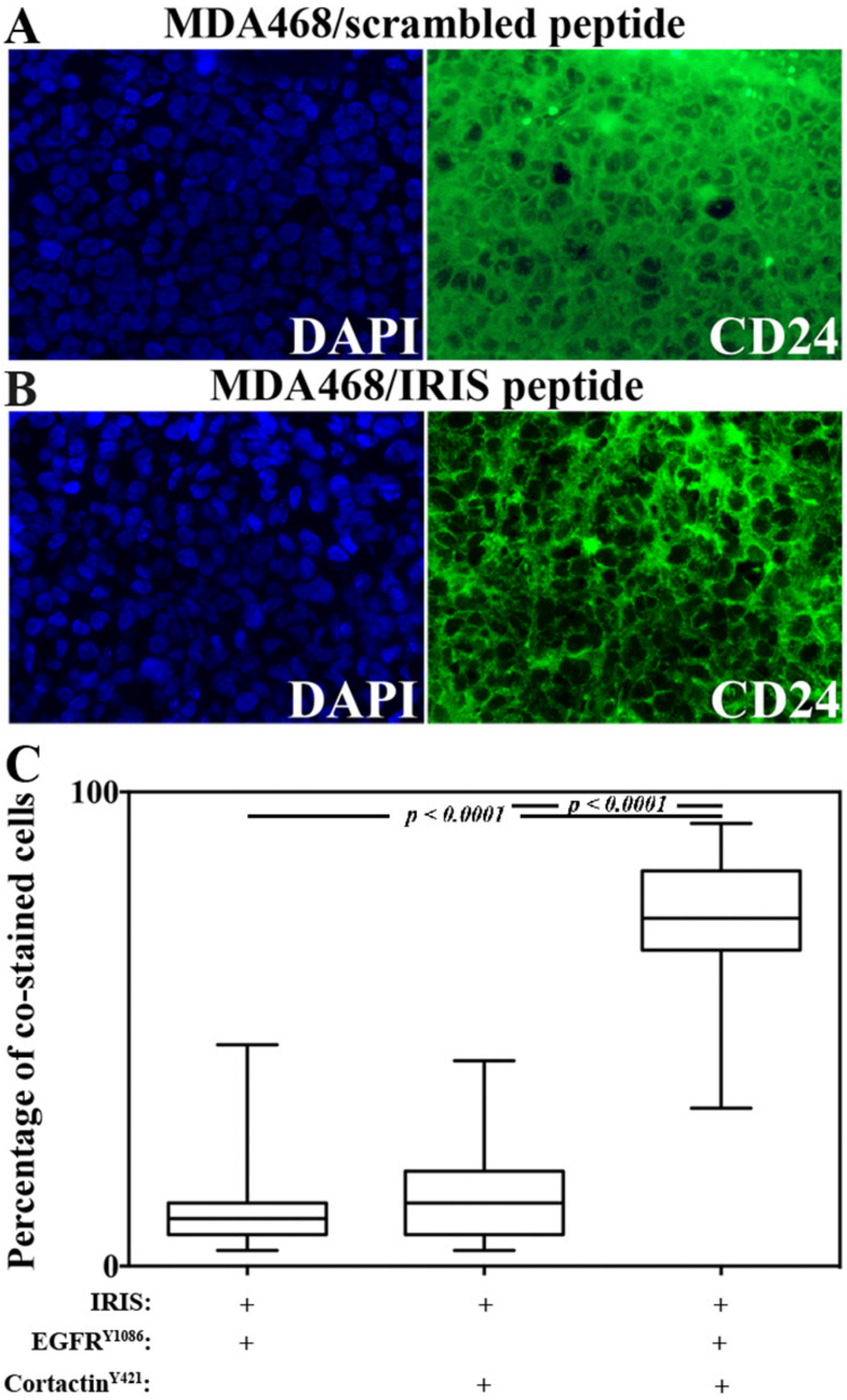

Supplementary Figure 3 (related to Figure 4): A. Predominantly c-CD24 expression as detected using fluorescence IHC in scrambled peptide treated MDA468 orthotopic mammary tumors developed in Nu/Nu mice. **B.** Predominantly m-CD24 expression in IRIS-inhibitory peptide treated MDA468 orthotopic mammary tumors developed in Nu/Nu mice. **C.** Percentage of cells co-stained with IRIS and p-EGFR^{Y1086} and/or p-cortactin^{Y421} in a cohort of TNBC (n=72) tumors.

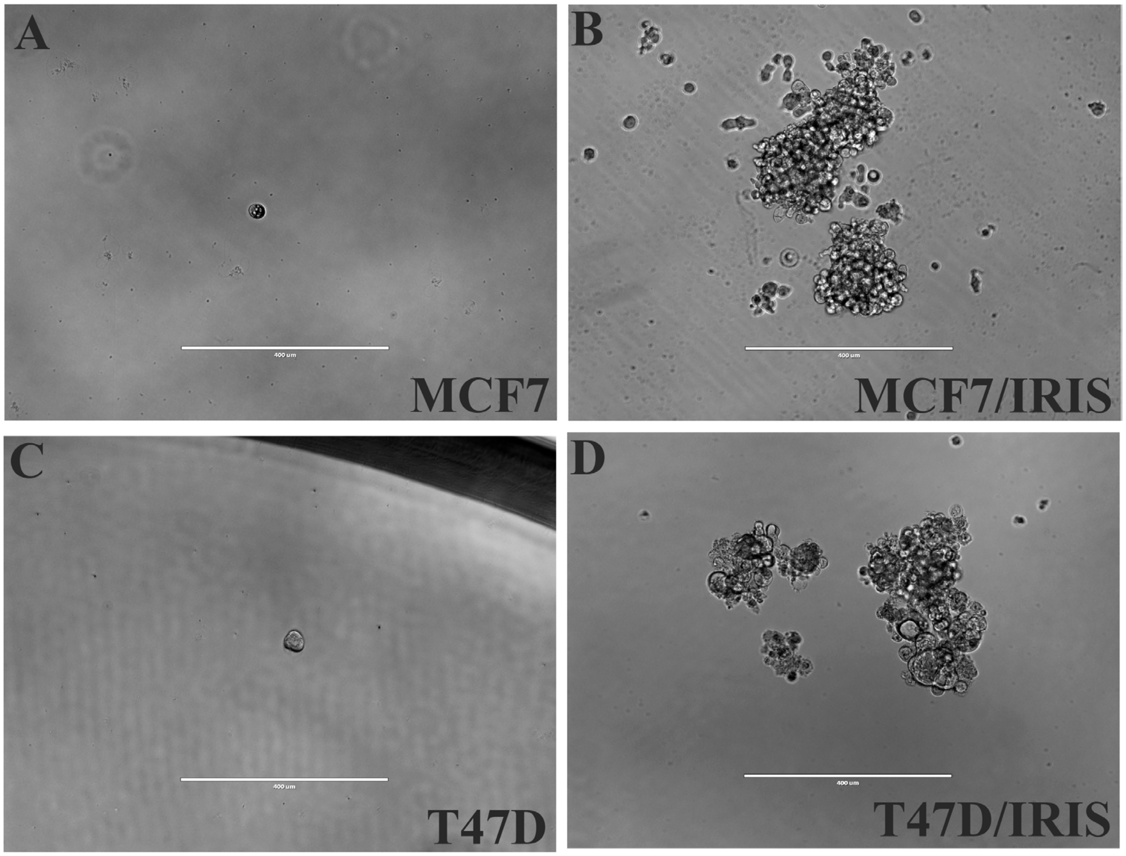

Supplementary Figure 4 (related to Figure 5): Mammosphers formation by parental **A.** or IRISOE **B.** MCF7 cells, and parental T47D **C.** or IRISOE **D.** T47D cells at 2 weeks.

	Total	IRIS-negative	IRIS-positive	*P* value
**Total number of cases**	N (%)	N (%)	N (%)	
	72 (100)	13 (18.0)	59 (82.0)	
**Metastasis status**				
**In situ**	2 (2.8)	0 (0.0)	2 (3.3)	0.507073
**Localized**	42 (58.3)	9 (69.2)	33 (55.9)	
**Lymph node**	22 (30.6)	4 (30.8)	18 (30.5)	
**Distant metastasis**	6 (8.3)	0 (0.0)	6 (10.3)	

Supplementary Figure 5 (related to Figure 6): The metastatic profile in a cohort of TNBC patients (n=72) with tumors expressing normal (IRIS-negative) or overexpressing (IRIS-positive) tumors.

Supplementary Table 1:

Antibody	Catalogue no.	Source
Cytokeratin 5	ab75869	Abcam Inc. (Cambridge, MA, USA)
Cytokeratin17 (EPR1624Y)	ab51056	Abcam Inc.
gamma Tubulin	ab11320	Abcam Inc.
anti-P cadherin/ CDH3 (6A9)	ab19350	Abcam Inc.
CD24-PE (SN3)	ab77219	Abcam Inc.
Twist (Twist2C1a)	ab50887	Abcam Inc.
CD44-FITC (IM7)	ab19622	Abcam Inc.
p-Cortactin (Y421)	ab196038	Abcam Inc.
EGFR (D38B1)	no. 4267	Cell Signaling Inc. (Danvers, MA, USA)
p-EGFR (Y1068) (D7A5)	no. 3777	Cell Signaling Inc.
p-Src (Y416)	no. 6943	Cell Signaling Inc.
Slug (C19G7)	no.9585	Cell Signaling Inc.
Sox2 (D6D9)	no. 3579	Cell Signaling Inc.
N cadherin/CDH2	no. 610920	BD Biosciences, (San Jose, CA, USA)
Cortactin	no. 610049	BD Biosciences
Oct3/4	MAB1759	R&D Systems (Minneapolis, MN, USA)
Nanog	Af1997	R&D Systems
CD24 (FL-80)	sc-11406	Santa Cruz Biotechnology Inc. (Dallas, TX, USA)
BRCA1 (Ab-2)	OP93	EMD Millipore (Billerica, MA, USA)
Actin	cp01	Calbiochem (San Diego, CA, USA)
Vimentin/LN6 (Ab-1)	IF01	Calbiochem
ALDH1/ALDH1A1	AM1846B	ABGent

*Mouse anti-human BRCA1-IRIS was developed in our laboratory (see Chock et al., BRCA1-IRIS overexpression abrogates UV-induced p38MAPK/p53 and promotes proliferation of damaged cells. 2010; 29(38):5274-5285).

Supplementary Table 2:

Gene	Primer sequence
FOXC2	Forward 5`-GCCTAAGGACCTGGTGAAGC-3`<br>Reverse 5`-TTGACGAAGCACTCGTTGAG-3`
E-cadherin	Forward 5`-TGCCCAGAAAATGAAAAAGG-3`<br>Reverse 5`-GTGTATGTGGCAATGCGTTC-3`
N-cadherin	Forward 5`-ACAGTGGCCACCTACAAAGG-3`<br>Reverse 5`-CCGAGATGGGGTTGATAATG-3`
Fibronectin	Forward 5`-CAGTGGGAGACCTCGAGAAG-3`<br>Reverse 5`-TCCCTCGGAACATCAGAAAC-3`
Vimentin	Forward 5`-GAGAACTTTGCCGTTGAAGC-3`<br>Reverse 5`-GCTTCCTGTAGGTGGCAATC-3`
Snail	Forward 5`-CCTCCCTGTCAGATGAGGAC-3`<br>Reverse 5`-CCAGGCTGAGGTATTCCTTG-3`
Twist	Forward 5`-GGAGTCCGCAGTCTTACGAG-3`<br>Reverse 5`-TCTGGAGGACCTGGTAGAGG-3`
SLUG	Forward 5`-GGGGAGAAGCCTTTTTCTTG-3`<br>Reverse 5`-TCCTCATGTTTGTGCAGGAG-3`
Sox2	Forward 5`-TTCATCGACGAGGCTAAGCGGCTG-3`<br>Reverse 5`-AGCTGCCGTTGCTCCAGCCGTTCA-3`
Oct4	Forward 5`-ACATGTGTAAGCTGCGGCC-3`<br>Reverse 5`-GTTGTGCATAGTCGCTGCTTG-3`
Nanog	Forward 5`-ATGCCTCACACGGAGACTGT-3`<br>Reverse 5`-AGGGCTGTCCTGAATAAGCA-3`
CK5	Forward 5`-GCGGTTCCTGGAGCAGCAGAACAAGGTTCT-3`<br>Reverse 5`-CTGAGGTGTCAGAGACATGCGTCTGCATCT-3`
CK17	Forward 5`-CTGGCTGCTGATGACTTCCGCACCAAGTTT-3`<br>Reverse 5`-CGCAGTAGCGGTTCTCTGTCTCCGCCAGGT-3`
CDH3	Forward 5`-CCATGGCAACAAAGAGCAGCTGACGGTGAT-3`<br>Reverse 5`-CAATTATAAAGTTGCCGATTTCATCTGGGA-3`
EGFR	Forward 5`-CCAGGACCCCCACAGCACTGCAGTGGGCAA-3`<br>Reverse 5`-GTGGGTGTAAGAGCTAATGCGGGCATGGCA-3`
β-catenin	Forward 5`-CCTGTACGCCAACACAGTGC-3`<br>Reverse 5`-ATACTCCTGCTTGCTGATCC-3`
hIRIS	Forward 5` GTCTGAGTGACAAGGAATTGGTTT 3`<br>Reverse 5` TTAACTATACTTGGAAATTTGTAAAATGTG 3`
GAPDH	Forward 5` AATGGAAATCCCATCACCATCT 3`<br>Reverse 5` CGCCCCACTTGATTTTGG 3`
